# Forty Years Since the Epidemic: Modern Paradigms in HIV Diagnosis and Treatment

**DOI:** 10.7759/cureus.14805

**Published:** 2021-05-02

**Authors:** Karan Patel, Alex Zhang, Michelle H Zhang, Sean Bunachita, Basil M Baccouche, Henna Hundal, Liseth K Lavado, Aakshi Agarwal, Preeti Malik, Urvish K Patel

**Affiliations:** 1 Medicine, Cooper Medical School of Rowan University, Camden, USA; 2 Psychological & Brain Sciences and Biology, Johns Hopkins University, Baltimore, USA; 3 Molecular and Cellular Biology, Johns Hopkins University, Baltimore, USA; 4 School of Medicine, Stanford University, Stanford, USA; 5 Max Bell School of Public Policy, McGill University, Montreal, CAN; 6 Nursing, Rutgers School of Nursing, Newark, USA; 7 Biology, Yale University, New Haven, USA; 8 Public Health, Icahn School of Medicine at Mount Sinai, New York, USA; 9 Neurology, Massachusetts General Hospital, Boston, USA; 10 Public Health and Neurology, Icahn School of Medicine at Mount Sinai, New York, USA

**Keywords:** hiv, hiv aids, aids, hiv drugs, hiv treatment, hiv diagnosis

## Abstract

Human immunodeficiency virus (HIV) is a viral infection that, when transmitted through the exchange of certain bodily fluids, destroys various immune cells and contributes to an overall weakened immune system. If left untreated, HIV progresses to acquired immunodeficiency syndrome (AIDS) - a chronic, life-threatening condition that puts patients at risk for opportunistic infections. Since the emergence of HIV nearly a century ago, the world has seen tremendous advances in elucidating its pathology and progression. These advances have been accompanied by an increased understanding of how subsequent effects and symptoms manifest in afflicted individuals. These discoveries, coupled with the ever-improving technologies and methodologies used for detection and treatment, provide the scientific and medical community with a solid grasp of HIV. Despite this significant headway, there is still much progress to be made; medical advances have allowed people with HIV to manage their disease and live a longer, healthier life, but a definite cure is yet to be found. Thus, the following literature review serves as both an extensive compendium of our current understanding of HIV - its pathology, testing/detection, repercussions, and treatment - and an acknowledgement of the areas that still require further research.

## Introduction and background

It is thought that human immunodeficiency virus (HIV) originated in Kinsahi in the Democratic Republic of Congo in the 1920s, arising as a result of cross-species infection from chimpanzees to humans. It was not until the 1980s, though, that the virus gained global traction when two groups of gay men in the United States were concurrently infected with various rare diseases. In Los Angeles, one group of men was infected with *Pneumocystis carinii* pneumonia - now renamed *Pneumocystis jiroveci* pneumonia - while at the same time, another group in New York was infected with an aggressive form of Kaposi sarcoma. Within a decade, the virus rapidly spread to 145 countries, leading to 400,000 cases worldwide [1]. Throughout the decades, as scientists began uncovering the mechanism underlying HIV infection, therapeutic approaches were developed to significantly reduce HIV-associated mortality and infection. However, HIV continues to persist throughout the world. As of 2019, 36.2 million adults and 1.8 million children have a current HIV diagnosis, and on average, there have been 1.7 million new infections annually [2]. Although HIV can infect a person of any age group, the rates of infection are the highest among people between the ages of 25 and 29. Although it is thought that the primary form of transmission is through intercourse, it can also be spread through other means such as intravenous drug use [3].

## Review

Human immunodeficiency virus structure

HIV is part of a family of viruses known as the *Retroviridae*. As an enveloped virus, HIV contains both a capsid and an envelope that is formed through budding and sequestration of the human lipid bilayer. The HIV genome is composed of two single-stranded RNA molecules with three important sections termed gag, pol, and env. These sections also code for corresponding protein products that are vital to the HIV lifecycle. The gag gene section codes for four proteins that are important to the capsid of HIV: p24, p17, p7, and p6. These proteins serve to form the conical capsid, solidify the inner membrane layer, form the nucleoprotein and RNA complex, and facilitate virus particle release. Notably, the pol gene codes for p10, p51, and p32, which correspond to protease, reverse transcriptase, and integrase, respectively. These proteins perform enzymatic functions that are crucial to HIV survival and replication [4]. Finally, the env section of the HIV genome contains two important proteins called gp120 and gp41 that appear on the envelope of HIV. These proteins help facilitate attachment and entry of HIV into host cells upon binding to the corresponding receptor [5].

Mechanism of human immunodeficiency virus infection

The mechanism of HIV infection is well understood. To start, once HIV enters a host, it uses its gp120 envelope protein (a viral attachment protein) to bind to a CD4 receptor on a T cell. Additionally, a third variable loop region on the gp120 protein allows it to bind to a coreceptor on the T cell. This coreceptor can either be CCR5 (more common), CXCR4, or both. Generally, during the early stages of infection, CCR5 is the main coreceptor, but as the infection progresses, gp120 binds to either CCR5 or CXCR4, or switches completely over to CXCR4. Certain drugs, such as CCR5 inhibitors, can also cause the virus to evolve and become CXCR4-tropic [6]. After binding to the receptor and fusing to the membrane, the viral RNA makes its way inside of the cell, where reverse transcriptase converts it into DNA. This is then incorporated into the host cell’s DNA using an integrase enzyme. The viral particles are then synthesized from newly integrated viral DNA using the host cell’s machinery. Finally, after the viral particles are made and assembled, they exit the cell through exocytosis, egressing with a part of the host cell membrane as their new envelope. The protease enzymes cleave newly synthesized viral proteins at various cleavage sites in order to create a fully mature HIV virion, capable of infecting a new cell [7,8].

Tropism testing

Although clinicians generally assume HIV infections use the CCR5 receptor as an entry point, there are tests that can be conducted to determine which coreceptor is used. These tests can be divided into two categories: genotypic and phenotypic. Genotypic tests rely on sequencing the HIV strain and looking primarily at the V3 loop in the gp120 envelope protein. The significant positions within this protein loop are the amino acid residues at positions 11, 24, and 25. If these amino acids are basic residues, then the envelope protein has a net positive charge and the HIV virus is considered CXCR4-tropic [9]. On the other hand, if these positions lack basic residues, then the virus is considered CCR5-tropic. In comparison, phenotypic methods are far more complicated and laborious; such methods involve creating vectors with the HIV envelope protein and a luciferase indicator. After placing the virus among T cells, the luciferase indicator lights up CXCR4, CCR5, or both depending upon which receptors the virus binds to. These tests are known as either trofile or trofile ES - a newer version with higher sensitivity and specificity [10].

Effects of human immunodeficiency virus

Viral Load

Viral load, along with CD4 count, is perhaps the most important prognostic factor in both disease manifestations and patient outcomes. In the absence of antiretroviral therapy (ART), initial viral load and CD4 count serve as a way to estimate how likely a patient is to progress from HIV to acquired immunodeficiency syndrome (AIDS) (classically defined as a CD4 count of <200). A viral load of <500 copies/mL along with a CD4 count of >750 in an adult patient means that there is only a 3.6% chance that the patient will progress to AIDS in a nine-year time frame. On the other hand, an initial viral load of >30,000 copies/mL with a CD4 count of >500 puts a patient at a 76.3% chance of progressing to AIDS within nine years. During the acute HIV infection, the viral load in a patient spikes due to a lack of a mature immune response [11]. During the latent part of an HIV infection, the viral load comes back down, remains at a set point, and then spikes again toward the end of the infection (usually marking death of the patient). Viral load determination is done using real-time polymerase chain reaction (PCR) that takes advantage of SYBR green intercalating fluorescent dye, which can measure fluorescence when viral DNA is amplified. The greater the quantity of initial viral DNA, the larger the initial fluorescence signal will be, and the earlier maximum fluorescence will be reached. The fluorescence data from the patient sample can then be compared to a standard curve to determine a patient’s viral load [12].

CD4 Count

CD4 cells serve as conductors of the immune system. They release a wide array of cytokines that include, but are not limited to, interleukin 2 (IL2), IL4, IL5, IL10, TGFB, and interferon gamma (INFγ), which cause the response of the innate immune system, B cell proliferation, and the response of natural killer cells, CD4, and CD8 cells. Because HIV attacks CD4 cells - untreated HIV can reduce a patient’s CD4 cell count between 50-60 cells per year - a patient becomes more susceptible to other infections [13]. Flow cytometry is used to determine CD4 cell count. This method is based on the diffraction of laser light in the forward direction, known as forward scatter (used to measure cell size) and sideways deflection known as side scatter (used to measure cell complexity). Each subpopulation of cells has a different amount of forward and side scatter and, as a result, can be differentiated based on those properties. Before putting the cells through a flow cytometry machine, they are bound to fluorescent antibodies. These antibodies bind to receptors, such as the CD4 and CD8 receptors, and function to help differentiate T cells. When the cells are passed through the flow cytometer, the machine not only detects the forward and side scatter of the laser light but also recognizes whether a fluorescent antibody is bound to a cell. Critically, the fluorescent antibodies give off different colors of light based on the subpopulation of T cells that they are bound to. For example, a fluorescent antibody that is bound to CD4+ T cells may give off green light, while another population of antibodies that is bound to CD8+ T cells may give off red light. Through this method, subpopulations of cells can be counted as well, although here we focus on CD4 cells as they are most pertinent to HIV infection [14]. In a normal patient the ratio of CD4 to CD8 cells is about 2:1. However, in an HIV-infected patient, the ratio can undergo as drastic a change as flipping to become approximately 2:1 for CD8 to CD4 cells, respectively, although patients can experience a variety of ranges. In general, if the ratio of CD4 to CD8 cells falls under 1, there is reason to raise suspicion of an HIV infection [15,16].

Acquired immunodeficiency syndrome/Opportunistic Infections

An opportunistic infection is an infection that occurs in an individual with an impaired immune system. These infections do not normally occur in healthy individuals, even with exposure to the etiology of the disease. These diseases can stem from environmental pathogens, reactivation of old diseases, or infections through constantly prevalent viruses [17]. AIDS is one of the most well known causes for a weakened immune system. AIDS results from a prolonged HIV infection that depletes a patient’s CD4 count over time. An average healthy adult will have somewhere between 500-1,200 cells/mL of CD4 cells. In contrast, AIDS is typically defined through a CD4 count of less than 200 cells/mL. However, there has been an expanded criteria for the diagnosis of AIDS to include: (1) CD4 < 200 cells/mL, (2) pulmonary tuberculosis, (3) recurrent bacterial pneumonia (greater than or equal to two episodes a year), (4) invasive cervical cancer, and (5) presentation with an AIDS-defining opportunistic infection [18].

Human immunodeficiency virus testing

Fourth-Generation Testing

The primary form of testing used today is a fourth-generation HIV test. While other methods such as PCR are available and can deliver a result faster, they are not widely employed for financial reasons. A fourth-generation test detects an HIV infection within two weeks and has a specificity of >99.8% and a sensitivity of 99.5% [19].

Fourth-generation tests determine if a patient has HIV by looking for a combination of the antibodies toward gp120, found on the outer envelope of the HIV virus, and the presence of the gp24 antigen, found in the viral capsid. In order to test for both of these methods, an enzyme-linked immunosorbent assay (ELISA) is employed. The gp120 antibody test uses an indirect ELISA, whereas the gp24 antigen test uses a sandwich ELISA. In the gp120 antibody test, gp120 is first fixed at the bottom of a plate or well before the patient’s serum is poured over the plate. If the patient’s sample has the gp120 antibody, it binds to the gp120 antigen that is already fixed to the bottom of the plate. Next, an anti-human gp120 antibody coupled to an enzyme is added, which binds to the human gp120 antibody if it is present and bound to the gp120 antigen. Finally, a substrate is added to the mixture that reacts with the coupled enzyme to change color. A greater intensity of color change corresponds to a larger degree of antibody present in the serum of the patient. One of the disadvantages of this method is the delay in diagnosis as antibodies against gp120 are made after p24 antigen is already present in the blood. As a result, researchers developed a sandwich ELISA to the p24 antigen to develop a method of testing that would allow for earlier diagnoses. In the sandwich ELISA, the bottom of the well is fixed with an antibody for the gp24 antigen of the virus. Then, the patient’s serum is poured over the well. If any p24 antigen from the virus is present, it binds to the antibodies. Finally, another antibody, this time conjugated to an enzyme, is placed into the solution. This creates a scenario in which a present gp24 antigen is bound by two antibodies. Again, a substrate is added such that, if the gp24 antigen is present, the antibody bound to the enzyme causes a color change in the solution [20,21].

If the fourth-generation test comes back positive, additional testing is performed. According to the algorithm designed by the Centers for Disease Control and Prevention, a positive test then defaults to determining which type of HIV infection a patient is infected with (HIV-1 or HIV-2) by examining the patient’s antibodies. While this is relatively straightforward, there are a couple of outcomes that can complicate the diagnosis and require additional testing. In some cases, the antibodies are determined to be negative for both HIV-1 and HIV-2 or indeterminate. In this case, a nucleic acid test, usually PCR, is done in order to determine whether the patient actually has an HIV-1/2 infection or if the first result was a false positive [22]. 

Third and Fifth-Generation Testing 

Before the development of fourth-generation testing, the primary mode of detecting an HIV infection was determining if a patient had antibodies to the virus (third-generation testing). In contrast, current fourth-generation tests detect both antigen and antibody, rather than antibody alone. This form of testing allows an HIV diagnosis to be made approximately one week earlier as viral antigen presents in the bloodstream before the patient’s antibodies do [19,23]. Today, fifth-generation tests are available, and use the same mechanism as fourth-generation tests, but are an advancement in that they can differentiate between the presence of the gp24 antigen or the gp120 antibody [19]. This test has a higher sensitivity of 100% and the same specificity of 99.5%, but still takes approximately two weeks from an acute HIV infection to make a positive diagnosis [19]. Although fourth-generation tests cannot make this distinction, they remain the most commonly used test.

Rapid Testing

Initially, one of the primary issues with HIV testing was the time it took to receive a confirmation of the diagnosis. Test results had to be sent to a lab where an ELISA was run - a process that could take several days. One issue that arises from this then is patients who come in for a test but do not return to receive their results. In order to speed up the process and alleviate similar problems, rapid HIV tests were developed. These tests utilize a lateral chromatography method that relies on antibodies and a fluid sample from the patient to detect the presence of HIV. While these tests are not as accurate as laboratory-based assays, they still have relatively high negative predictive value. If a test comes back positive, a second rapid test is performed in order to confirm the diagnosis [24].

Pediatric human immunodeficiency virus

Since the onset of HIV in the United states, there have been a total of 15,000 children infected and approximately 3,000 HIV-related pediatric deaths [25]. Although the number of infections is declining, children born to HIV-positive mothers still present many challenges. First, because maternal antibodies transfer across the placenta and children born to HIV-infected mothers need to undergo PCR testing rather than traditional antibody tests. In order for a child to be considered HIV-free, they must have two negative PCR tests conducted when they were over one month old [26]. Another challenge that arises with pediatric HIV is that CD4 counts are markedly different in children than they are in adults, and so adult guidelines for immunocompromised states cannot be applied. Moreover, dosing regimens have to be adjusted based on age-specific approvals, and following the strict dosing guidelines can be challenging in pediatric populations.

Vertical transmission of HIV can occur during three separate time periods: antenatal, intrapartum, and postpartum. Antenatal transmission can occur in utero through transplacental passage of HIV [27]. The intrapartum period refers to the time of labor and delivery. During this period, the fetus can become infected through exposure to the vaginal fluids and maternal blood. In order to prevent this, cesarean sections are recommended when the mother has a viral load greater than 1,000 copies/mL. These have been shown to reduce the transmission of HIV by up to 50% [28]. The postpartum period refers to the time after a child is born. The primary mode of postpartum transmission occurs through breastfeeding. In developed countries, such as the United States, it is recommended that an HIV-positive mother does not breastfeed a child, regardless of her viral load and CD4 count. However, in developing countries, the guidelines still recommend an HIV-positive mother to exclusively breastfeed for the first six months, followed by a six-month period of solid food mixed with breastfeeding as long as she is on ART [29].

Human immunodeficiency virus drugs

The first HIV medication, zidovudine (AZT), first came out in 1987. At the time, clinical trials showed that taking the medication beginning at 14 weeks gestation, intravenously during labor, and six months postpartum reduced vertical transmission of HIV by up to 67% [30]. Since then there have been many HIV drugs that have been developed and various combinations of drugs that have been employed. After the advent of AZT, researchers noticed that there were rapidly occurring mutations to AZT, and so a two-drug regimen was recommended to try to combat HIV resistance [31]. However, resistant forms of HIV quickly sprung up in the face of two-drug combinations, resulting in the employment of three-drug combinations [32]. Fortunately, three-drug combinations have been shown to be extremely effective and are part of the current recommended guidelines [33]. It should be noted, however, that although three-drug regimens are extremely effective, a patient must strictly adhere to the daily dosing regimen otherwise the virus will rapidly mutate [34]. While adherence to a one-pill medication does not seem difficult on a surface level, it can be extremely challenging for certain groups of populations such as the homeless or those with neurological issues such as dementia or amnesia.

The drugs that have been developed thus far all pay homage to scientists having uncovered the mechanism of HIV entry into a cell. The various stages and enzymes (receptor binding, fusion, reverse transcriptase, integrase, and protease) of viral entry have served as the primary targets of treatments and therapies [35]. In the following paragraphs, we describe the mechanism behind each drug class and list common medications and major side effects to each of the medications in the various classes.

Mechanisms of Action of Drug Classes

Each step of the mechanism of HIV infection can serve as a potential target for therapeutic drugs. CCR5 inhibitors (Table 1) work by binding to the CCR5 receptor and preventing attachment of HIV to the receptor. Fusion inhibitors (Table 2) work primarily by binding to the gp41 protein of the HIV envelope. For HIV to infect a cell, the gp41 protein must undergo a conformational change to aid in entry. By binding to the gp41 envelope, these classes of drugs prevent the conformational change from occurring and therefore inhibit entry. Nucleoside reverse transcriptase inhibitors (NRTIs) (Table 3) are the most widely used class of HIV drugs. These drugs mimic DNA nucleotides but lack a three-prime hydroxyl group, which prevents chain elongation when added into the growing DNA strand. NRTIs work by binding to the hydrophobic pocket of the reverse transcriptase and causing a conformational change, which renders the enzyme inactive. Non-nucleoside reverse transcriptase inhibitors (NNRTIs) (Table 4) bind to the p66 domain of the reverse transcriptase enzyme and causes it to undergo a conformational change which leads to inhibition of enzyme activity. Protease inhibitors (Table 5) work through molecular mimicry: they resemble the structure of normal peptides that would be cleaved by the protease enzyme [35]. Integrase inhibitors (Table 6) function by interfering with divalent cations (usually magnesium), which are necessary for function of the active site of the enzyme. While there are more drugs on the market than the ones below, we focused only on the ones that are currently used in clinical settings. The latest guidelines for drug regimens recommend to use a three-drug regimen with two NRTIs and a single other drug from any of the following categories: NRTIs, protease inhibitors, and integrase inhibitors (this is currently the drug class of choice). In order to make treatment adherence more convenient for patients, combination drugs have been created where two NRTIs and one integrase inhibitor is integrated into one pill to be taken once a day [33].

Note that the limitations listed below are not a comprehensive list, rather we focused on the ones that are most clinically relevant.

**Table 1:** CCR5 inhibitors.

Drug name	Limitations
Maraviroc	Cannot be taken as part of a single drug regimen. Can also cause abdominal pain, orthostatic hypotension, and musculoskeletal symptoms [6].

**Table 2:** Fusion inhibitors.

Drug name	Limitations
Enfuvirtide	The primary mode of delivery is through injections. These injections can lead to the development of painful nodules. Also, these cannot be taken as a single drug regimen. May also lead to hypersensitivity reactions (in 1% of patients) and neutropenia [36].

**Table 3:** Nucleoside reverse transcriptase inhibitors. HIV: human immunodeficiency virus

Drug names	Limitations
Zidovudine	Used perinatally in order to reduce transmission risk for HIV-infected mothers to children; however, long-term use is associated with macrocytic anemia. Can also cause myopathies presenting as proximal muscle tenderness [37,38].
Tenofovir Disoproxil Fumarate	Can cause renal toxicity and decreases in bone mineral density [10].
Tenofovir Alafenamide	Improved version of Tenofovir Disoproxil Fumarate. Relatively well-tolerated with few side effects [39].
Abacavir	Required to have Hla-b 5701 testing done in patients as it may cause hypersensitivity reactions, including a rash with multiorgan system involvement, fever, nausea, and vomiting [37].
Emtricitabine	Relatively well-tolerated with few side effects. Can cause hypopigmentation of palms and soles [36].
Lamivudine	Relatively well-tolerated, but has the potential to cause hepatitis B flair when medication is stopped [36].

**Table 4:** Non-nucleoside reverse transcriptase inhibitors.

Drug names	Limitations
Rilpivirine	Has to be taken with a 400-calorie meal and cannot be used if the patient's viral load is >100,000 particles/mL or CD4 <200. Can also cause rash, dizziness, and adrenal insufficiency. High doses may result in a prolonged QTc interval [40-42].
Doravirine	This drug is relatively well-tolerated in patients; however, it is not used as part of the recommended guidelines because it has not undergone an inferiority trial comparing it to the integrase inhibitors (primary drugs of choice) [43].
Efavirenz	Causes neuropsychiatric issues in many patients, including insomnia, drowsiness, and vivid dreams [36].

**Table 5:** Protease inhibitors. HIV: human immunodeficiency virus

Drug names	Limitations
Atazanavir	Causes jaundice which can lower adherence. Can also cause kidney stones and abdominal pain [44].
Darunavir	Has the highest barrier to resistance out of all the HIV drugs. Contains a sulfa moiety; however, there have been relatively few adverse reactions [45].

**Table 6:** Integrase inhibitors.

Drug names	Limitations
Dolutegravir	Increases serum creatinine levels by 0.1-0.2 mg/dL. Can also cause drug-related insomnia [39].
Bictegravir	Increases serum creatinine levels by 0.1-0.2 mg/dL [39].
Raltegravir	Can cause rhabdomyolysis and myopathy, and is dosed twice daily. Can also cause drug-related insomnia [39].

Resistance Mutations

As mentioned above, the general course of treatment for HIV is one combination pill that includes two NRTIs and one integrase inhibitor. While this triad is generally extremely effective, some patients develop drug-resistant HIV mutants, and therefore, their course of treatment will have to be altered. The major sign that raises clinical suspicion for HIV mutations is when a patient whose CD4 count and viral load begin to increase in the aftermath of the period of decline that accompanies initial treatment. Similar to tropism testing, there are two major ways to test for drug susceptibility: phenotypic and genotypic. The phenotypic method begins when a researcher uses a viral vector made up of the PCR fragments of a patient’s gag/protease/reverse transcriptase and infects a T cell in vitro. Then, varying concentrations of drugs are added to the culture dish, and the concentration of the drug required to control the infection is measured. These concentrations are measured against a standard table that has various concentrations of drugs required to kill a non-mutant form of the virus. A ratio of concentration of drug used to kill the mutant virus divided by concentration of drug used in normal virus is calculated. A ratio of one would imply that the mutant virus form is just as susceptible to the drug as the standard HIV virus, while greater than one would imply that the mutant virus has greater resistance to the drug [46]. There is a standard reference that determines how high the ratio must be for the mutant virus to be considered resistant to the drug. Genotypic methods are far less labor-intensive and can allow for faster results. These methods involve sequencing the patient’s HIV strain, and then predicting resistance to drugs based on the sequence. There are various databases that allow for comparison of the patient’s HIV sequence to sequences with known resistance to various drugs. The main drawback of this method of using the database is that it is an indirect measurement of drug resistance and novel mutations may not be present in databases [47].

## Conclusions

Although the incidence of HIV, both in the United States and globally, is decreasing, we still face many challenges associated with the disease. One of the main challenges with preventing the spread of HIV is that many people are not aware of their infection due to a prolonged asymptomatic period. This leads to asymptomatic spreading of the virus and unmonitored transmission. Ideally, clinicians should diagnose the infection as early as possible in order to get a person on ART and prevent viral spread, but this can be difficult with asymptomatic individuals who do not feel the need to see a healthcare provider. Thus, ongoing efforts to increase awareness and promote consistent asymptomatic testing are crucial. Additionally, there remains a deep-rooted stigma associated with HIV which can discourage people from disclosing their status for fear of isolation and loss of community support. It is clear that, even though we have made tremendous progress over the last half century, we still have a ways to go before the end of the HIV era.
